# Genetic Diversity of *Babesia canis* Strains in Dogs in Lithuania

**DOI:** 10.3390/microorganisms10071446

**Published:** 2022-07-18

**Authors:** Jana Radzijevskaja, Dalytė Mardosaitė-Busaitienė, Asta Aleksandravičienė, Birutė Karvelienė, Miglė Razgūnaitė, Inga Stadalienė, Algimantas Paulauskas

**Affiliations:** 1 Faculty of Natural Sciences, Vytautas Magnus University, K. Donelaičio Str. 58, LT-44248 Kaunas, Lithuania; dalyte.mardosaite-busaitiene@vdu.lt (D.M.-B.); asta.aleksandraviciene@vdu.lt (A.A.); migle.razgunaite@vdu.lt (M.R.); algimantas.paulauskas@vdu.lt (A.P.); 2 Faculty of Veterinary Medicine, Lithuanian University of Health Sciences, Tilžės Str. 18, LT-47181 Kaunas, Lithuania; birute.karveliene@lsmuni.lt (B.K.); inga.stadaliene@lsmuni.lt (I.S.)

**Keywords:** *Babesia canis*, canine babesiosis, PCR-RFLP, 18S rRNA, Bc28.1, Lithuania

## Abstract

Canine babesiosis is an emerging and rapidly expanding tick-borne disease in central and northeast Europe. In the last two decades, the endemic area of *Babesia canis* has expanded from central Europe to the Baltic region. This study aimed to investigate the genetic diversity of *B. canis* strains isolated from naturally infected dogs in different regions of Lithuania using PCR-RFLP and sequence analyses based on a partial region of 18S rRNA and *Bc28.1* genes. Blood samples from 149 dogs suspected of having babesiosis were collected in Lithuania during 2016–2017. Based on PCR-RFLP profiles and two nucleotide substitutions observed in 18S rRNA gene sequences, three *B. canis* genotypes were identified in Lithuania—18S rRNA-A, 18S rRNA-B and 18S rRNA-A/B—with the A/B genotype predominating (83.9%). Based on the obtained PCR-RFLP profiles of the Bc28.1 gene, four *B. canis* genotypes were identified: Bc28.1-B (53.8%), Bc28.1-34 (20.8%), Bc28.1-A (17.9%), and Bc28.1-34/A or B (7.5%). Sequence analysis of the partial *Bc28.1* gene revealed eighteen polymorphic sites and thirteen sequence variants among the Lithuanian samples. The *B. canis* genotypes obtained were detected with varying prevalences in different regions of Lithuania.

## 1. Introduction

Canine babesiosis is an emerging tick-borne disease in dogs found worldwide that is caused by haemoprotozoan *Babesia* parasites, which are divided into two groups (small and large babesias) based on the size and morphology of intraerythrocytic forms [1]. Members of both groups are known to cause disease in canines (large-sized *Babesia canis*, *Babesia rossi* and *Babesia vogeli* and small-sized *Babesia gibsoni*, *Babesia conradae* and *Babesia microti*-like) [2].

The vast majority of clinical babesiosis cases in European dogs are caused by *B. canis* [3]. The clinical signs associated with *B. canis* vary from mild transient illness to acute disease, potentially leading to mortality. Canine babesiosis can be classified as complicated (with a high mortality rate) and uncomplicated (with low mortality rates). Complicated forms with a high mortality rate have mostly been observed in central Europe [4].

Canine babesiosis is an emerging and rapidly expanding infectious disease in central and northeast Europe [5]. The endemic area of *B. canis* has expanded from central Europe to the Baltic region, and new endemic foci of canine babesiosis have been documented in Germany, Poland, Lithuania and Latvia [6,7,8,9]. The spread of *B. canis* to new areas is closely connected to the expansion of the range of its main vector, the *Dermacentor reticulatus* tick [10,11,12]. In the last decade, *D. reticulatus* has expanded its range in Baltic countries. In Lithuania, *D. reticulatus* has been detected in new localities in which this species had not been previously reported. In Latvia, the presence of *D. reticulatus* has been confirmed in some of the southern regions [13] and subsequently in western and central Latvia, including the Riga region, indicating that the northern border of *D. reticulatus* in Europe has moved northwards [14]. In Lithuania, *Babesia* spp. was detected in 1.2% (26 of 2255) of *D. reticulatus*, whose prevalence in different locations varied from 0% to 11% [15]. In Latvia, *B. canis* was detected in *D. reticulatus* ticks with a prevalence of 0.34% [14].

Canine babesiosis is a major veterinary problem in Lithuania. The first cases of the disease were registered in the central part of the country in 2003. An increasing number of cases with a wide variety of clinical signs have been recorded in different regions since 2010 and canine babesiosis has become endemic throughout the country. The State Food and Veterinary Service of Lithuania does not hold statistics on the annual incidence of complicated and uncomplicated clinical babesiosis cases or record mortality among the Lithuanian dog population. Between 2011 and 2021, veterinary practitioners in various small animal clinics in central (Kaunas), eastern (Vilnius) and western (Klaipėda) parts of Lithuania documented between 20 and 150 cases of babesiosis per year, with the highest number of cases detected in central regions of the country. The Small Animal Clinic (Faculty of Veterinary Medicine, Lithuanian University of Health Sciences) in Kaunas documented 188 autochthonous canine babesiosis cases in 2019–2021: the incidence of canine babesiosis in 2019 was 59 cases per year and 8.6/1000 dogs, in 2020 it was 74 cases per year and 10.5/1000 dogs, and in 2021 it was 55 cases per year and 7.1/1000 dogs. In the first half of 2022, 28 babesiosis cases were diagnosed in dogs brought to this veterinary clinic.

Over the past few years, with increasing information about the prevention of canine babesiosis, some veterinarians have observed a declining trend in the incidence of the disease, especially its complicated forms [16]. Microscopic evaluation continues to be the most accessible diagnostic test for most veterinarians in Lithuania. The first study on the detection and molecular characterisation of *B. canis* strains based on sequence analysis of a partial region of 18S rRNA gene was conducted in Lithuania in 2014 [7]. The results of the study confirmed that *B. canis* is the etiological agent of the disease in dogs in the central part of Lithuania, and demonstrated the necessity of using molecular analysis for an accurate diagnosis of canine babesiosis (in cases when symptoms are weak or unspecific and blood smears cannot provide sufficient diagnostic information to veterinarians).

*Babesia canis* strains are distinct based on their virulence, antigenic differences and chromosome size polymorphism [17]. The difference in the virulence of *B. canis* strains is associated with observed genetic heterogeneity among *B. canis* isolates. In the last few decades, Pirodog^®^ (Merial, S.A.S., Lyon, France) and Nobivac^®^ Piro vaccines have been developed and are commercially available in Europe. Although dogs can be vaccinated against canine babesiosis, the level of protection is highly variable, which may be due to the genetic diversity of *B. canis* strains.

*Babesia canis* strains have been classified based on polymorphism in 18S rRNA and *Bc28.1* genes [17,18]. The 18S rRNA gene is relatively conserved and demonstrates very low genetic variation among *B. canis* strains, which limits the usefulness of this marker in studies of intraspecific diversity.

The *Bc28.1* gene belongs to multigene families composed of polymorphic genes [19]. This gene encodes a major protein from the *B. canis* merozoite surface, a 28 kDa GPI (glycosyl-phosphatidylinositol) anchored protein, which is involved in initial attachment to canine erythrocytes. Members of the *Bc28* gene family exhibit a high degree of intra-species antigenic variation and have been shown to be potential virulence factors. They have also recently attracted attention as candidates for innovative vaccine strategies and diagnostics [20,21]. Genetic variability and antigenic variation are considered to be important mechanisms in the survival of *Babesia* parasites in their vertebrate hosts [17]. The *Bc28.1* gene is currently used as a genetic marker for the evaluation of the genetic diversity of *B. canis* strains and their distribution in different European regions.

The aim of this study was to investigate the genetic diversity of *B. canis* strains isolated from naturally infected dogs in Lithuania using PCR-RFLP and sequence analyses based on a partial region of 18S rRNA and *Bc28.1* genes.

## 2. Materials and Methods

**Figure 1:**
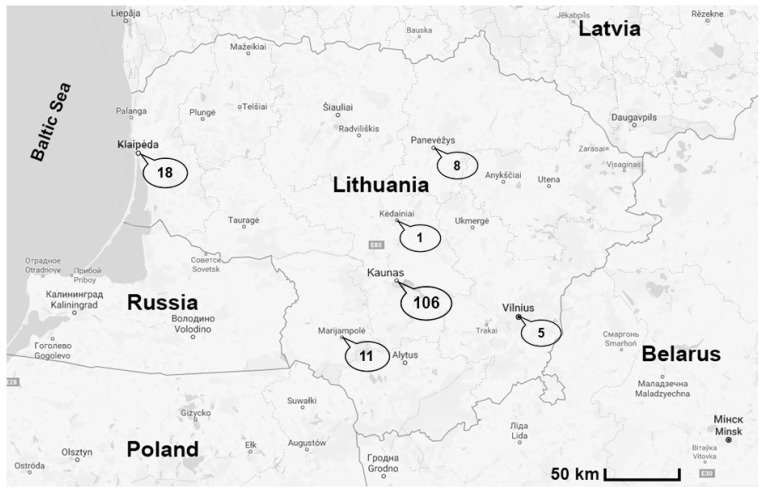
Map of sample collection sites. Blood samples were collected from 149 dogs originating from six regions of Lithuania. The number in the circle indicates the number of dogs analyzed.

During the seasonal spring outbreak of babesiosis in 2016–2017, 149 blood samples were collected from dogs of various breeds and ages with a presumptive diagnosis of babesiosis. The disease was diagnosed based on clinical signs and the examination of blood smears. The samples were collected from different veterinary clinics in six regions of Lithuania (Figure 1). The clinicopathological findings in *Babesia* infections were slight to severe thrombocytopenia, severe immune-mediated haemolytic anaemia (IMHA), cerebral babesiosis and acute kidney injury (AKI).

### 2.1. DNA Extraction

Genomic DNA was extracted from 200 μL aliquots of EDTA blood (taken from the vena cephalica of the examined dog) using the GeneJet Whole Blood Genomic DNA Purification kit (Thermo Fisher Scientific, Vilnius, Lithuania), according to the manufacturer’s instructions.

### 2.2. PCR-RFLP Analysis

Partial sequences from 18S rRNA and *Bc28.1* genes of *B. canis* were amplified. Conventional PCR was performed using primers BAB GF2 and primers BAB GR2, which amplify a 559 bp region of the 18S rRNA gene of *B. canis* [18]. A 710 bp fragment of the *Bc28.1* gene was amplified with F281&2a and R281 primers, as described in Carcy et al. [17]. Both PCR reactions were carried out in a 25 µL final volume containing 5 X MyTaq DNA buffer, 10 pmol of each primer, 5 U Taq DNA polymerase (Thermo Fisher Scientific, Vilnius, Lithuania), double-distilled water and 5 µL of DNA template. Amplification reaction of partial 18S rRNA gene was performed as follows: initial denaturation at 92 °C for 2 min, 50 cycles of denaturation at 92 °C for 60 s, annealing at 52 °C for 60 s and extension at 72 °C for 90 s and a final extension step at 72 °C for 5 min. PCR conditions for *Bc28.1* gene were initial denaturation at 94 °C for 3 min, followed by 35 cycles of denaturation at 94 °C for 30 s, annealing at 55 °C for 30 s and extension at 72 °C for 60 s. The final extension was at 72 °C for 7 min. In each PCR run, negative (double-distilled water) and positive (DNA of *Babesia* positive ticks, infection confirmed by sequencing) controls were used.

Positive 18S rRNA gene PCR products were digested with the *HincII* restriction enzyme [18] allowing the classification of *B. canis* isolates into two genotypes: 18S RNA-A, where the PCR fragment (559 bp) was cut into two fragments of 409 and 150 bp, and 18S RNA-B, where the 559 bp PCR fragment remained uncut (Figure S1).

PCR fragments of the *Bc28.1* gene were digested with the combination of two restriction enzymes (*MboI* and *AluI*) allowing the classification of *B. canis* isolates into three genotypes: Bc28.1-A, Bc28.1-B and Bc28.1-34 [17]. Genotype Bc28.1-A was identified after *MboI* digestion when the 710 bp PCR fragment was cut into 300, 290, 70 or 50 bp fragments (Figure S2). *AluI* digestion enabled the identification of strains from genotype Bc28.1-B, resulting in 320, 240, 90 and 60 bp fragments and genotype Bc28.1-34 into 410, 240 and 60 bp fragments (Figure S3).

A 2% agarose gel was prepared by visualising the results after the PCR-RFLP analysis in the ultra-violet transilluminator GelDoc-It 310 and VisionWorks LS (Ultra-Violet Products Ltd., Cambridge, UK). DNA fragment sizes were assessed by comparison with the GeneRulerTM 50 bp DNA Ladder (Thermo Fisher Scientific, Vilnius, Lithuania).

### 2.3. Sequence Analysis

Representative PCR products of 62 samples were extracted from the agarose gel and purified using the GeneJET Gel Extraction Kit (Thermo Fisher Scientific, Vilnius, Lithuania) according to the manufacturer’s instructions and sent for sequencing (Macrogen, Amsterdam, The Netherlands).

The obtained sequences were edited using the Mega X software package, aligned with each other, and compared with the sequence data available from GenBank using the NCBI BLAST^®^ software (http://blast.ncbi.nlm.nih.gov, accessed on 30 March 2022). A phylogenetic tree based on *B. canis Bc28.1* gene sequences was created using the neighbour-joining method with bootstrap analysis of 1000 replicates. The most appropriate model of nucleotide substitution determined according to the Bayesian information criterion was the Jukes-Cantor model, with a gamma distribution of among-site variation (JC + G). Partial 18S rRNA and *Bc28.1* gene sequences for representative samples were submitted to GenBank under accession numbers MN078319 to MN078323 for the 18S rRNA gene and MN078324 to MN078336 for the *Bc28.1* gene.

## 3. Results

### 3.1. Babesia canis 18S rRNA Genotypes

After amplification of the partial 18S rRNA gene, *B. canis* DNA was detected in 112 (75.2%) samples. All these samples were subjected to digestion with the *HincII* restriction enzyme. A selected number of amplicons (*n* = 30) derived from samples collected in different Lithuanian regions were sequenced to identify the molecular heterogeneity of different isolates (Table 1).

Based on the RFLP profiles obtained and two nucleotide GA/AG substitutions observed in 18S rRNA gene sequences, three *B. canis* genotypes were identified. Of these genotypes, a “mixed” genotype predominated that showed PCR-RFLP profiles specific to both 18S rRNA-A and 18S rRNA-B genotypes (Figure S1) and the presence of both G and A nucleotides (R/R) at positions 92 and 93 (corresponding to 610 and 611 nucleotides in the whole-length ssrRNA gene) (Table 1). A total of three single nucleotide polymorphisms in the partial 18S rRNA gene of *B. canis* and five different sequence variants were identified by sequence analysis (Table 1).

**Figure 2:**
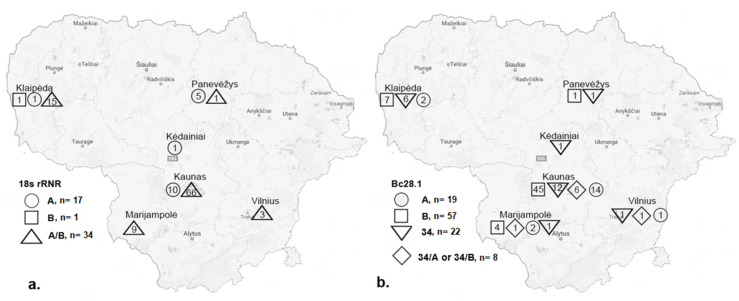
Distribution of *B. canis* 18S rRNA (**a**) and Bc28.1 (**b**) genotypes in Lithuania. On the map, different geometric shapes (circle, square, triangle, rhombus) with numbers represent the number of *B. canis* strains corresponding to each genotype found in different locations. *n*—the total number of *B. canis* strains of a particular genotype.

The predominant 18S rRNA-A/B genotype was detected in 94 (83.9%) blood samples from dogs and in all investigated regions of Lithuania except Kėdainiai. The *B. canis* genotype 18S rRNA-A was detected in seventeen (15.2%) dogs from Kaunas, Klaipėda, Kėdainiai and Panevėžys, while the *B. canis* genotype 18S rRNA-B was detected in just one dog (0.9%) in Klaipėda (Figure 2a).

### 3.2. Babesia canis Bc28.1 Genotypes

After amplification of the partial region of *Bc28.1* gene, *B. canis* DNA was detected in 106 (71.1%) samples. All these samples were subjected to digestion with the combination of two restriction enzymes (*MboI* and *AluI*). A selected number of amplicons (*n* = 35) derived from samples collected in different Lithuanian regions were sequenced (Table 2).

Based on the RFLP profiles obtained, three genotypes were identified: Bc28.1-B (57/106; 53.8%), Bc28.1-34 (22/106; 20.8%) and Bc28.1-A (19/106; 17.9%). Some analysed samples showed specific profiles of Bc28.1-34 and Bc28.1-A or Bc28.1-B (8/106; 7.5%) when the PCR fragment was cut into 410, 320, 240, 90 and 60 bp fragments (possibly co-infection) (Figures S2 and S3).

Sequence analysis of the 631 bp fragment of the *Bc28.1* gene revealed 18 polymorphic sites (15 nucleotide substitutions and 3 deletions) in the analysed samples (Table 2).

Analysis of the 210 amino acids of the Bc28.1 protein sequences revealed amino acid substitutions in 10 positions: 26 (aspartic acid to asparagine), 28 (lysine to glutamine), 50 (glutamic acid to lysine), 109 (glutamic acid to aspartic acid), 110 (aspartic acid to asparagine), 113 (lysine to asparagine), 149 (leucine to valine), 156 (lysine to asparagine), 173 (threonine to asparagine) and 204 (serine to asparagine). All other nucleotide changes were silent (Table 2).

Based on two observed nucleotide substitutions, G/A (150 position) and C/T (477 position), four genotypes were distinguished in accordance with the genotypes obtained by PCR-RFPL analysis [17] (Table 2).

*Bc28*.1 gene sequences of *B. canis* strains obtained in Lithuania were 99–100% identical to the corresponding sequences from laboratory *B. canis* strains obtained in France [17]. Lithuanian *B. canis* strains identified as belonging to Bc28.1-A genotype were 100% identical to the *B. canis* laboratory strain A8 deposited in GenBank (Acc.No. CS019629) or differed from it by two nucleotides. Other Lithuanian *B. canis Bc28*.1 gene sequences that belonged to the Bc28.1-B and Bc28.1-34 genotypes differed from the corresponding sequences of the *B. canis* laboratory strain B (GenBank Acc.No. KP863713) by one to four nucleotides and three deletions and *B. canis* laboratory strain 34.01 (GenBank Acc.No. KP863714) by three to five nucleotides, respectively (Table 2).

Five *Bc28*.1 gene sequence variants within the *B. canis* Bc28.1-34 genotype, four sequence variants within the Bc28.1-B genotype and two sequence variants within the Bc28.1-A genotype were detected. In four samples, G nucleotide was observed at position 150, which is characteristic of the Bc28.1-34 genotype, and T nucleotide was observed at position 477, which is characteristic of the Bc28.1-A genotype. Therefore, these *B. canis* strains have been described as belonging to the “mixed” Bc28.1-34/A genotype. Two different sequence variants were detected within this genotype (Table 2).

**Figure 3:**
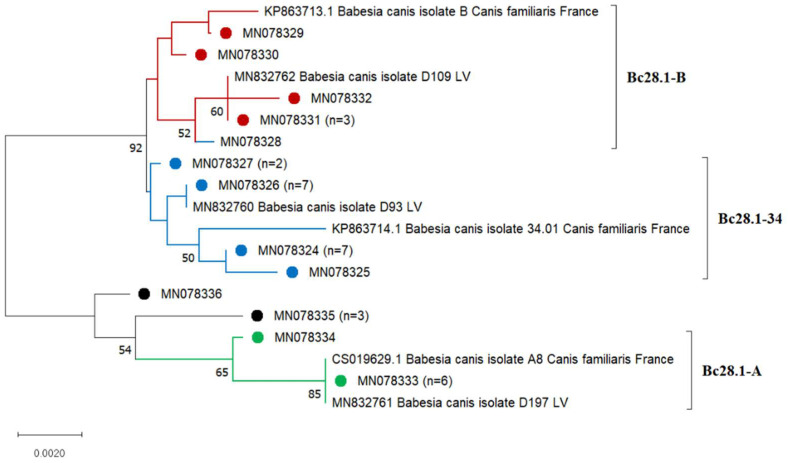
Phylogenetic tree of the *Bc28.1* gene sequences of *Babesia canis* created using the neighbour-joining method and Jukes-Cantor model with a gamma distribution of among-site variation. Bootstrap values (values < 50% not shown) from 1000 replicates are shown at the nodes. Samples sequenced in the present study are marked. The number of samples represented by the sequence is given in parentheses (*n* = x). *Babesia canis* strains corresponding to each genotype are marked with different colours.Analysing the results of the PCR-RFLP analysis, we observed that most of the *B. canis* infections in dogs were associated with the Bc28.1-B genotype (Figure 2b). This genotype was mainly detected in Kaunas (45 of 56 samples) and was also found in Klaipėda, Marijampolė and Panevėžys. The Bc28.1-34 genotype was detected in all the regions in which samples were collected, with the greatest prevalence in Kaunas (12 out of 22 samples). The Bc28.1-A genotype also prevailed in Kaunas (14 of 19 samples) and was detected in three other regions: Klaipėda, Marijampolė and Vilnius (Figure 2b). All four genotypes were detected in central Lithuania (Kaunas) and the south-western (Marijampolė) part of the country.

The phylogenetic tree was created to include 35 sequences of the *Bc28.1* gene obtained in this study and the 6 corresponding sequences of *B. canis* isolates obtained from dogs in France (Acc.No. KP863713, KP863714, CS019629) and Latvia (Acc.No. MN832760, MN832761, MN832762) (Figure 3). Most of the analysed sequences in the phylogenetic tree were separated into three major clusters according to the Bc28.1 genotype: Bc28.1-A, Bc28.1-B and Bc28.1-34. Sequence MN078328 (identified as belonging to the Bc28.1-34 genotype) clustered with the Bc28.1-B genotype sequences. Four sequences of the mixed genotype 34/A grouped with the Bc28.1-A genotype sequences and formed a single cluster on the phylogenetic tree (Figure 3).

## 4. Discussion

The molecular characterisation of *B. canis* 18S rRNA and *Bc28.1* genes indicates the presence of genetically heterogenic *B. canis* strains in Lithuania. Molecular analysis of the 18S rRNA gene showed a separation of the sequences into three genotypes based on two single nucleotide polymorphisms. In Europe, four *B. canis* genotypes related to GA → AG nucleotides substitutions (GA, AG, AA, RR) are present [22,23]. In the present study, the vast majority of dogs (83.9%) were infected with the “mixed” 18S rRNA-A/B genotype (displaying the presence of both G and A nucleotides (R/R)), which was detected in all investigated regions of Lithuania except Kėdainiai (probably because there was only one sample analysed from this region) (Table 1; Figure 2a). In previous studies, the “mixed” genotype has been identified with a high prevalence in dogs in central Lithuania (65.8%) and in *D. reticulatus* ticks across the country (43.8%) [7,15]. A similar result on the dominance of the “mixed” 18S rRNA-A/B genotype in dogs has been reported in Latvia (91%; 39/43) [8] and Poland (87%) [24], and in ticks in Switzerland [23]. The presence of the ambiguous nucleotides detected in 18S rRNA sequences of *B. canis* isolates is explained by the genetic heterogeneity occurring among copies of the ss rRNA genes or by mixed infections with parasites of different 18S rRNA genotypes [22,23,24,25].

*B. canis* genotypes 18S rRNA-A (GA nucleotides) and 18S rRNA-B (AG nucleotides) occur in European countries at different rates of prevalence [17,26]. In the previous studies conducted in Lithuania, the 18S rRNA-A genotype was identified in 50% and 34.2% of *B. canis* sequences originating from *D. reticulatus* ticks and dogs, respectively [7,15]. The *B. canis* 18S rRNA-B genotype (AG nucleotides) is uncommon in Lithuania and has so far only been detected in one dog and one tick sample [15].

*Babesia canis* 18S rRNA-A and 18S rRNA-B genotypes were initially described in Poland by Adaszek and Winiarczyk [18]. Adaszek et al. [27] examined different strains of *B. canis* isolated from 76 dogs and found an association between the intensification of thrombocytopenia and particular 18S rRNA genotypes of the pathogen: the strains of *B. canis* genotype 18S rRNA-B were found to be more virulent in relation to thrombocytopenia (mean number of thrombocytes 61.11 × 109/L) than genotype 18S rRNA-A (mean number of thrombocytes 27.47 × 109/L). In this study, haematological data on thrombocytopenia was available for 40 dogs from Kaunas. Among these dogs, the *B. canis* 18S rRNA-A/B genotype was detected in 39 individuals and the 18S rRNA-A genotype in 1 dog. Thrombocyte counts below the lower reference range value (150 × 109/L) were detected in 28 (70%) dogs. Marked thrombocytopenia (thrombocyte counts lower than 27 × 109/L) has been found in 22 (55%) dogs (21 dogs with mixed A/B genotype and 1 with A genotype). Thrombocyte counts in the remaining dogs varied between 193 × 109/L and 488 × 109/L. As most infections were associated with the “mixed” 18S rRNA-A/B genotype, it was impossible to find a correlation between different 18S rRNA genotypes and the virulence of *B. canis* strains. It is believed that genotyping of *B. canis* strains based on the 18S rRNA gene is not suitable to determine the virulence of strains among 18S rRNA-A and B genotypes in regions where the “mixed” 18S rRNA-A/B genotype dominates.

Carcy et al. [17] first used the polymorphism of the *Bc28.1* gene to study the genetic diversity of *B. canis* in Europe. Based on PCR-RFLP and sequence analysis applied to four *B. canis* laboratory strains (A8, B, 34.01, and G) originating from France, three genetic groups—Bc28.1-A, Bc28.1-B and Bc28.1-34/G—were identified, which is in agreement with the classification of strains according to differences in chromosome profiles, virulence and antigenic variation. The *B. canis* B strain is more virulent in dogs than the *B. canis* A strain, while 34.01 isolate is genetically distinct from the *B. canis* isolates A and B on the basis of chromosome size polymorphism [28]. In the present study, clinicopathological findings were compared with *B. canis* genotype data, and no relationship was observed between the Bc28.1 genotype and the severity of the disease. Data on complicated and uncomplicated forms of canine babesiosis were recorded from 45 dogs at one Kaunas veterinary clinic. Eight (17.8%) dogs developed a complicated form of the disease. In these dogs, three Bc28.1 genotypes were detected: B (*n* = 4; 50%), A (*n* = 3; 37.5%) and 34 (*n* = 1; 12.5%). Uncomplicated babesiosis was diagnosed in 37 dogs, with the B genotype detected in 23 samples (62%), A in 5 samples (13.5%), 34 in 5 samples (13.5%) and 34/A in 4 samples (10.8%).

Carcy et al. [17] analysed *B. canis* isolates collected from dogs in nine European countries between 2002 and 2010. The distribution and prevalence of *B. canis* Bc28.1 genotypes differed between four European regions. In France, genotype B dominated (62.4%), followed by genotypes A (37.1%) and 34 (11.8%). In south-west Europe, genotype B predominated, while in north-east Europe it was genotype A. In central Europe, both genotypes (A and B) were found with a similar prevalence. Carcy et al. [17] identified genotype 34 only in the south of France (mostly as co-infection with genotypes A or B). This genotype is now found in Lithuania and Latvia [29]. The expansion of *D. reticulatus* during the last decade in Europe could favour the spreading of certain *B. canis* genotypes in new areas. The results of the present study show that the distribution and prevalence of *B. canis* Bc28.1 genotypes in Lithuania are specific and similar to findings in France but differ from those obtained in north-east and central Europe [17].

Different patterns in the prevalence and geographical distribution of *B. canis* Bc28.1 genotypes have been found in Lithuania’s neighbour, Latvia. In a recent study conducted by Kivrane et al. [29], the presence of three *B. canis Bc28.1* gene sequence types (Figure 2) with a non-uniform geographical distribution was detected. Based on the obtained results, the authors suggest two separate events in the establishment of *B. canis* foci in Latvia. Among the strains detected, predominant strains of the A genotype were mainly detected in Riga and the Riga region (Acc.No. MN832761). In the past few years, *D. reticulatus* had been detected in geographically separate new localities in the Riga region, indicating the development of new foci outside the major distribution area [14]. These recently established populations of *D. reticulatus* could favour the spread of *B. canis* strains of the A genotype in this region of Latvia. In Latvia, the *B. canis* strain of the B genotype (Acc.No. MN832762) was detected in just one sample obtained near the Lithuanian border [29], whereas, in Lithuania, this genotype was the most frequent. *B. canis* strains closely related to the 34.01 (Acc.No. MN832760) were detected in different regions of Latvia, mostly in the region of Daugavpils, which is relatively close to Lithuania and where the first populations of *D. reticulatus* ticks were detected [13]. No genetic variation was observed among sequences of A and 34 genotypes in the Latvian samples [29], which is in contrast to the findings of the present study. In Lithuania, five different sequence variants were detected within the *B. canis* 34 genotype, and two within the A genotype. It is noteworthy that all *Bc28.1* gene sequence variants obtained in Latvia were detected in the present study. These findings could indicate the recent expansion of *D. reticulatus* ticks from Lithuania and the establishment of particular *B. canis* strains in Latvia. However, other transmission routes from Belarus and Russia (where the *B. canis* Bc28.1-A genotype dominates [17]) are also possible.

Local ecological factors may play a crucial role in the survival of particular *B. canis* genotypes [17]. The emergence of *B. canis* is mainly associated with the expanding range of *D. reticulatus*, environmental changes, and migrations of humans and animals. In the past two decades, changes in climatic and ecological conditions, as well as the anthropogenic impact on the formation of the landscape, have made many areas in Baltic countries suitable for the expansion of animal hosts for all active stages of ticks and for the establishment of *D. reticulatus*, which has high ecological plasticity [13]. The geographical range of *D. reticulatus* in Europe is fragmented and divided into two main zones: western Europe and eastern Europe. A recent study investigated the *D. reticulatus* population structure through its distribution range in western and eastern Europe using microsatellite markers and demonstrated that this tick forms two genetically distinct groups across Europe (located in western and eastern Europe, with an overlap in central Europe) [30]. Genetically different *D. reticulatus* populations have been detected in the Czech Republic, Slovakia, western and eastern Poland, and Lithuania. Analysis of two mtDNA 12S rRNA and 16S rRNA markers has demonstrated that there are different haplotypes of *D. reticulatus* present in Europe ([31], Paulauskas et al., unpublished). This suggests that genetically different *D. reticulatus* populations could harbour different *B. canis* genotypes. Further studies are needed to analyse the relationship between *B. canis* strains, their geographical distribution and genotypes of *D. reticulatus*.

Co-infections of *B. canis* with other agents of canine vector-borne diseases could occur. Co-infected cases are of major clinical importance because they often lead to atypical clinical signs and abnormal laboratory findings, and may cause misdiagnosis and failures in treatment and prognosis [32]. Co-infections with *B. canis* and other vector-borne pathogens may result in greater pathogenicity and complications in the infected dogs. Blood samples from 70 dogs infected with *B. canis* (which were examined in this study) were analysed by real-time PCR for the presence of the other vector-borne pathogens transmitted by mosquitoes (*Dirofilaria repens*) and *I. ricinus* ticks (*Anaplasma phagocytophilum* and *Borrelia* spp.) [33], and double, triple or even quadruple co-infections in dogs were detected. These findings demonstrate that co-infections of *B. canis* with other vector-borne pathogens in dogs are to be expected in Lithuania and that simultaneous detection of multiple vector-borne pathogens in dogs is needed for accurate diagnosis of canine vector-borne diseases in the country.

## 5. Conclusions

The molecular characterisation of *B. canis* 18S rRNR and *Bc28.1* genes based on PCR-RFLP and sequence analyses indicated the presence of genetically heterogenic *B. canis* strains in Lithuania. The *B. canis* genotypes obtained were detected with different rates of prevalence in different regions of Lithuania. These findings provide a better understanding of the epidemiology of canine babesiosis and demonstrate the need for further investigation of the relationship between the genetic structure of *B. canis* parasites, their geographical distribution and strain virulence. Determination of the genetic diversity of the *Bc28.1* gene may be helpful for vaccine development and the assessment of its potential use in Baltic countries.

## Figures and Tables

**Table 1:** *Babesia canis* genotypes based on the comparison of 18S rRNA gene sequences. The number of samples represented by the sequence is given in parentheses (*n* = x).

Genotype	GenBank Accession Number (Sample Number)	Nucleotide Positions		
		80	92	93
A	MN078319 (*n* = 11)	T	G	A
	MN078322 (*n* = 2)	Y	.	.
B	MN078320 (*n* = 1)	.	A	G
A/B	MN078321 (*n* = 15)	.	R	R
	MN078323 (*n* = 1)	Y	R	R

**Table 2:** Comparison of *B. canis Bc28.1* gene sequences obtained from dogs in Lithuania with selected GenBank sequences. The number of samples represented by the sequence is given in parentheses (*n* = x).

Geootype	GenBank ace.No(Number of Samplcs)	Nucleotide Positions ^a,b^																			
		76	82	93	148	150	246	327	328	339	420	433	468	477	518	525	526	527	573	585	611
A	CS019629	G(D)	A(K)	G	G(E)	A	C	A(E)	G(D)	A(K)	C	C(L)	A(K)	T	C(T)	G	G	A	C	G	G(S)
	MN078333 (*n* = 6)	.	.	.	.	.	.	.	.	.	.	.	.	.	.	.	.	.	.	.	.
	MN078334 (*n* = 1)	.	.	.	.	.	.	C(D)	A(N)	.	.	.	.	.	.	.	.	.	.	.	.
B	KP863713	G	C(Q)	G	G	A	T	C	A	T(N)	A	C	T(N)	C	C	G	G	A	A	G	G
	MN078329 (*n* = 1)	.	.	.	.	.	.	.	.	.	.	.	.	.	A(N)	-	-	-	.	.	.
	MN078330 (*n* = 1)	.	.	.	.	.	.	.	.	.	.	.	.	.	A	-	-	-	C	.	.
	MN078331 (*n* = 3)	.	.	.	A(K)	.	.	.	.	.	.	.	.	.	A	-	-	-	C	.	.
	MN078332 (*n* = 1)	.	.	.	A	.	.	.	.	.	.	.	.	.	A	-	-	-	C	A	.
34	KP863714	A(N)	C	A	G	G	T	C	A	T(N)	A	G(V)	T	C	A	-	-	-	A	G	G
	MN078324 (*n* = 7)	G	.	.	.	.	.	.	.	.		C	.	.	.	-	-	-	C	.	.
	MN078325 (*n* = 1)	G	.	.	.	.	.	.	.	.		C	.	.	.	-	-	-	C	.	A(N)
	MN078326 (*n* = 7)	G	.	G	.	.	.	.	.	.		C	.	.	.	-	-	-	.	.	.
	MN078327 (*n* = 2)	G	.	G	.	.	.	.	.	.		C	.	.	.	-	-	-	C	.	.
	MN078328 (*n* = 1)	G	.	G	A	.	.	.	.	.		C	.	.	.	-	-	-	C	.	.
																					
34/A	MN078335 (*n* = 3)	G	C	G	G	G	C	A	G	A	C	C	A	T	A	-	-	-	A	G	G
	MN078336 (*n* = 1)	.	.	.	.	.	.	.	.	.	.	.	T	.	.	-	-	-	C	.	.

^a^—amino acid changes are represented in parentheses; ^b^—nucleotide and amino acid substitutions detected among Lithuanian strains are in bold. Gray-shaded columns indicate recognition sites for *Alu*I and *Mbo*I restriction enzymes based on which genotypes were identified.

## Data Availability

Data are contained within the article.

## Supplementary Materials

The following supporting information can be downloaded at: https://www.mdpi.com/2076-2607/10/7/1446/s1, Figure S1: Identification of *B. canis* genotypes using the *HincII* restriction enzyme. Lines M—Gene RulerTM 50 bp DNA ladder (Thermo Fisher Scientific Baltics, Lithuania); Line K−—negative control; Line K+—positive control; Line 1—genotype B; Lines 10, 12—genotype A; Lines 2–9, 11, 13–15—genotype A/B; Figure S2: Identification of *B. canis* genotypes using the *MboI* restriction enzyme. Lines M—Gene RulerTM 50 bp DNA ladder (Thermo Fisher Scientific Baltics, Lithuania); Line K−—negative control; Line K+—positive control; Lines 1–4, 6–10, 12–15—genotype B/34.01; Lines 5,11—genotype A; Figure S3: Identification of *B. canis* genotypes using the *AluI* restriction enzyme. Lines M—Gene RulerTM 50 bp DNA ladder (Thermo Fisher Scientific Baltics, Lithuania); Lines 1–8, 10–14—genotype Bc28.1-B; Line 16—genotype Bc28.1-34.01; Line 9—“mixed” genotype.
