# Supplementary material for: Genetic Diversity of Babesia canis Strains in Dogs in Lithuania
Source: Microorganisms. 2022 Jul 18;10(7):1446. doi: 10.3390/microorganisms10071446 (PMC9351669; doi:10.3390/microorganisms10071446)
Supplement: Supplementary file 1 [file microorganisms-10-01446-s001.zip › microorganisms-1822056-supplementary.pdf]

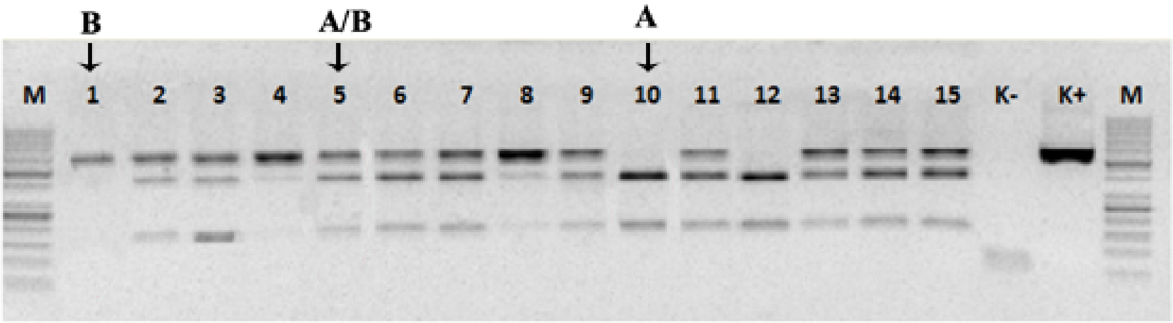

Figure S1: Identification of B. canis
genotypes using the HincII restriction enzyme. Lines M—Gene RulerTM 50 bp DNA ladder (Thermo Fisher Scientific Baltics, Lithuania); Line K⍰—negative control; Line K+—positive control; Line 1—genotype B; Lines 10, 12—genotype A; Lines 2–9, 11, 13–15—genotype A/B.

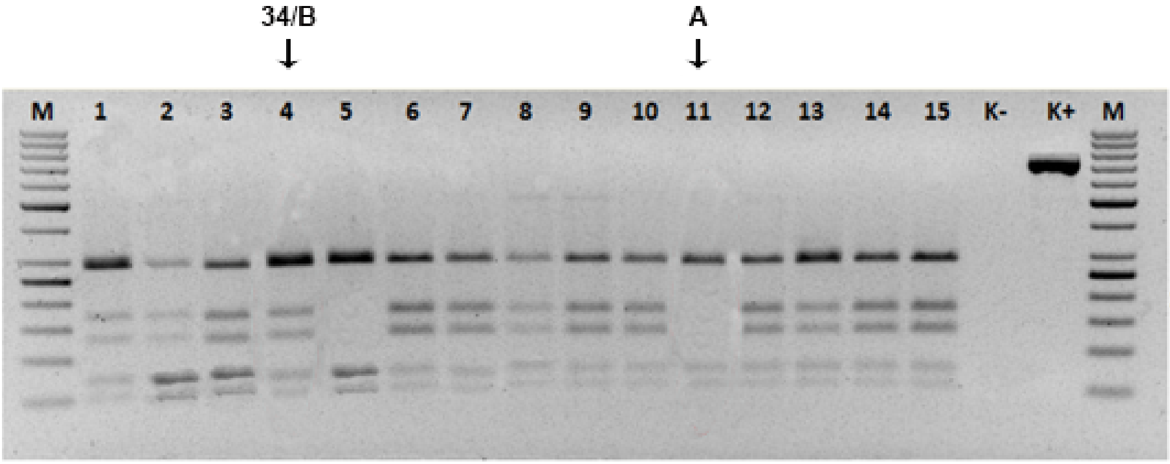

Figure S2: Identification
of B. canis genotypes using the MboI restriction enzyme. Lines M—Gene RulerTM 50 bp DNA ladder (Thermo Fisher Scientific Baltics, Lithuania); Line K⍰—negative control; Line K+—positive control; Lines 1–4, 6–10, 12–15—genotype B/34.01; Lines 5,11—genotype A.

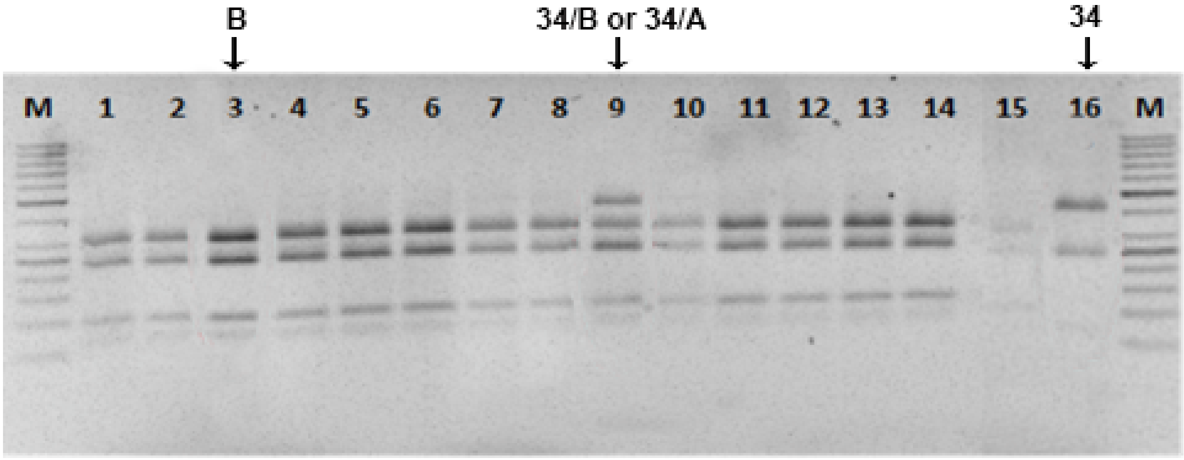

Figure S3: Identification of
B. canis genotypes using the AluI restriction enzyme. Lines M—Gene RulerTM 50 bp DNA ladder

(Thermo Fisher Scientific Baltics, Lithuania); Lines 1–8, 10–14—genotype Bc28.1-B; Line 16—genotype Bc28.1-34.01; Line 9—“mixed” genotype.
